# Supplementary material for: Cancer Stem Cell and Aggressiveness Traits Are Promoted by Stable Endothelin-Converting Enzyme-1c in Glioblastoma Cells
Source: Cells. 2023 Feb 3;12(3):506. doi: 10.3390/cells12030506 (PMC9914402; doi:10.3390/cells12030506)
Supplement: Supplementary file 1 [file cells-12-00506-s001.zip › cells-2135302-supplementary.pdf]

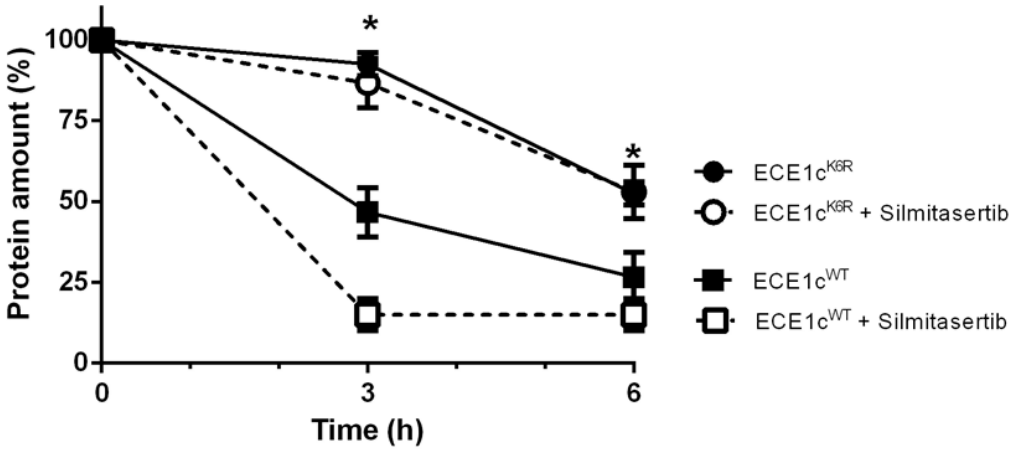

Figure S1: Pixel quantification plot of ECE1c levels from U87MG. Flag-tagged ECE1c^{WT} or ECE1c^{K6R}-expressing cells were treated with 20 µg/ml cycloheximide (CHX) in the absence or presence of 25 µM silmitasertib for 6 h. ECE1c protein levels were evaluated by Western blot with an anti-Flag antibody, using β-actin as loading control. Band pixels were quantified and plotted (*$p<0.05$).

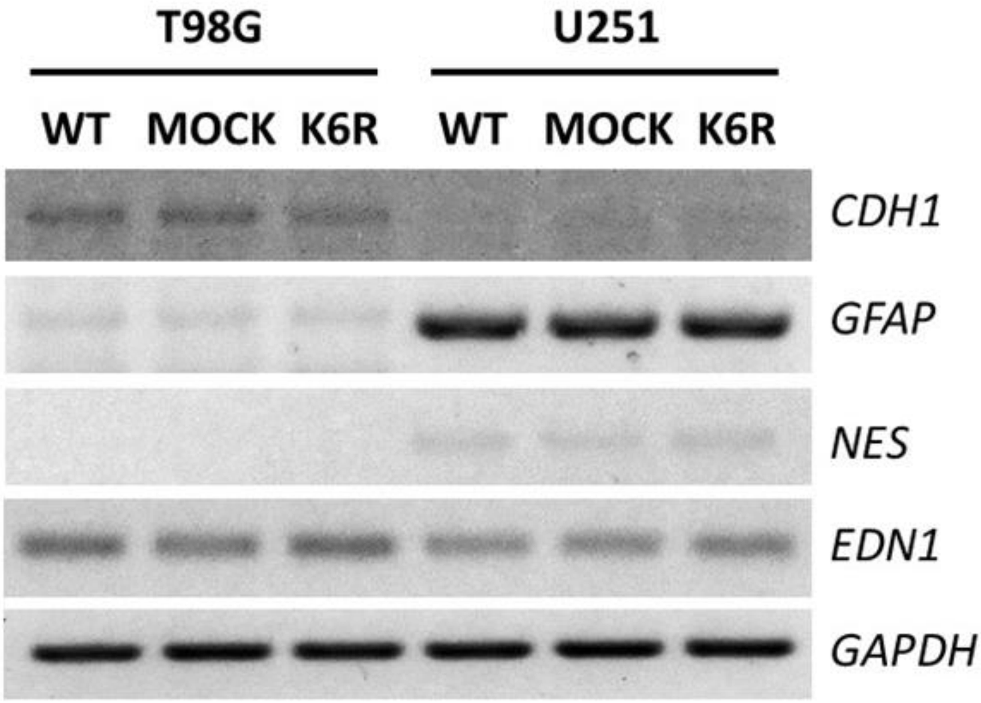

Figure S2: Transcript levels from ECE1c^{WT} or ECE1c^{K6R}-expressing cells (T98G and U251). Mock, ECE1c^{WT} or ECE1c^{K6R} GBM cell clones after 24 h under normal culture conditions were evaluated for mRNA levels of genes CDH1 (E-cadherin), GFAP (Glial fibrillary acidic protein), NES (Nestin), EDN1 (Endothelin-1) and GAPDH by conventional RT-PCR.

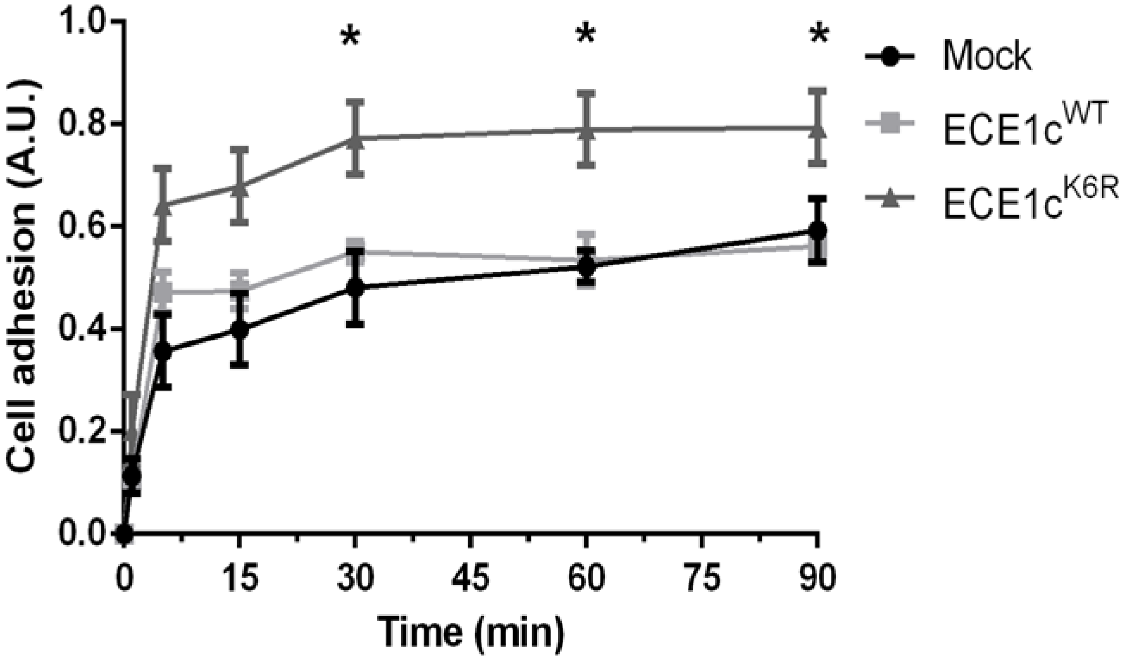

Figure S3: Cell adhesion of U87MG cells is enhanced under ECE1c^{K6R} overexpression. Mock, ECE1c^{WT} or ECE1c^{K6R}-expressing cells were seeded into 96-well plates pre-coated with 2 μg/ml fibronectin and cell adhesion was tested between 5-90 min. Cells were fixed, stained and color was measured and plotted for each cell line (*$p<0.05$).

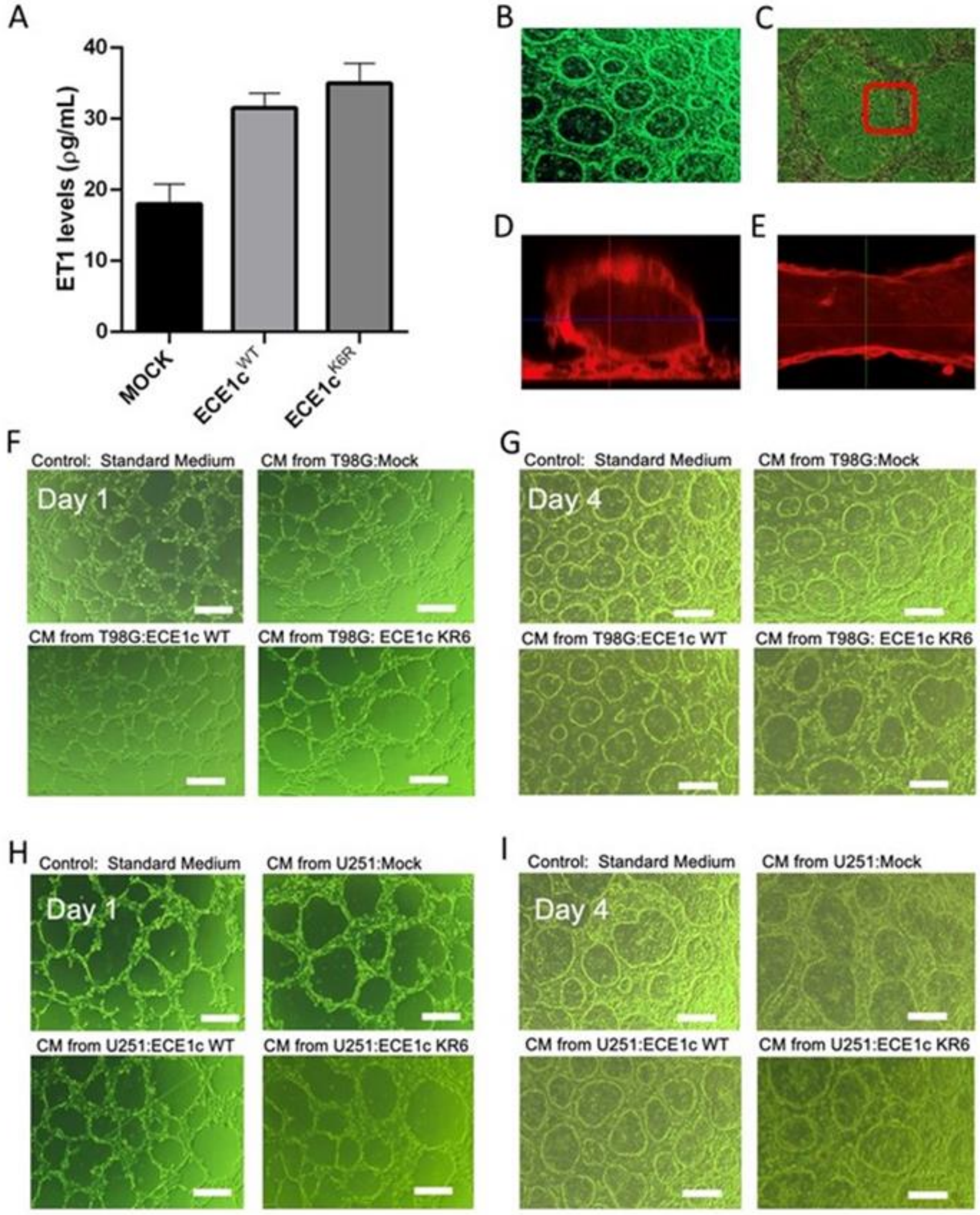

Figure S4: Endothelin-1 in conditioned media from GBM cells does not alter the capacity of HEY-A8 cells to undergo vasculogenic mimicry. (A) Conditioned media (CM) from mock, ECE1c^{WT}- and ECE1c^{K6R}-overexpressing GBM cells was analyzed by ELISA assay (Thermofisher EIAET1) to detect secreted ET1 levels. (B) HEY-A8 ovarian cancer cells form tubular structures over a monolayer of cells grown on Matrigel. (C) These structures stain positive (red) with Periodic Acid Schiff and highlight (within red square) the presence of a glycoprotein-rich lumen by confocal microscopy (D,E). CM from T98G and U251 cells allow the formation of initial intercellular connections at day 1 (F,H), followed by lumen-containing structures over a cell monolayer at day 4 (G,I). Images are representative of three independent experiments.

A

B

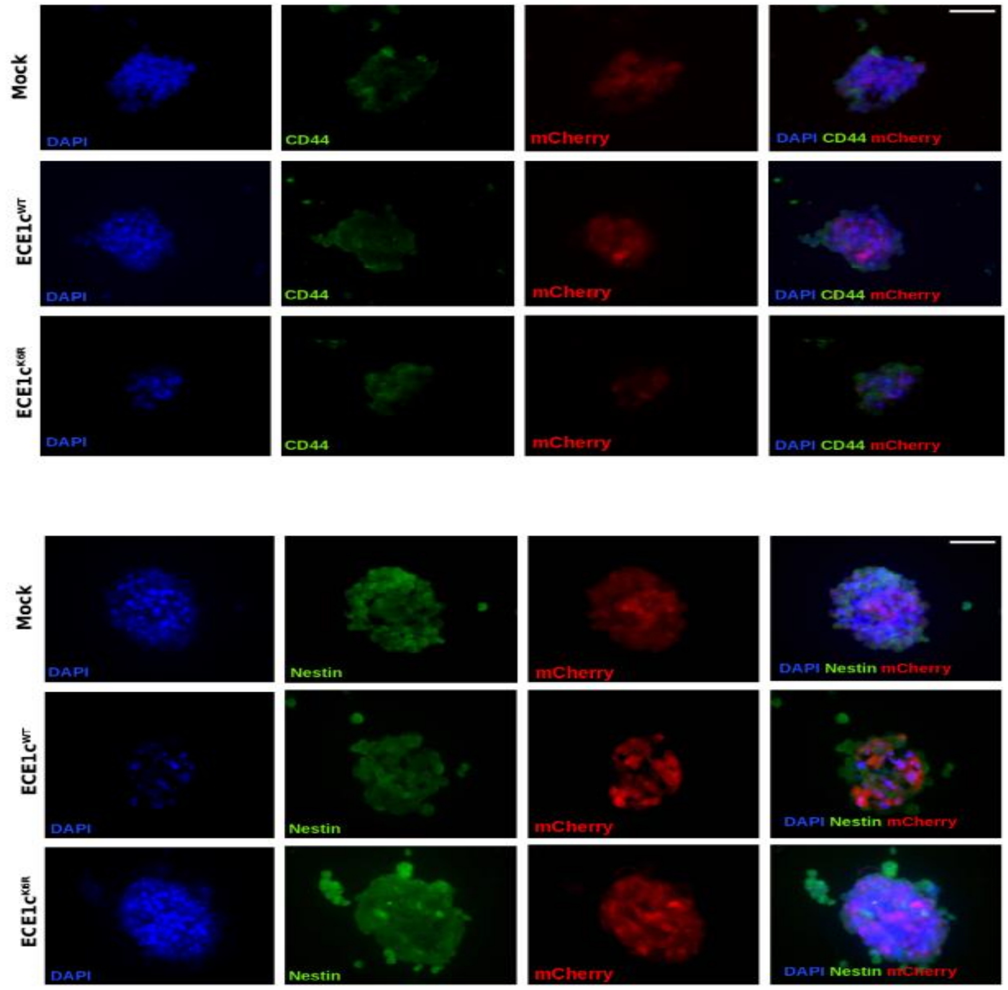

Figure S5: GSCs marker detection by IIF. GSCs enrichment was performed with U87MG clone cells expressing ECE1c^{WT}, ECE1c^{K6R} or mock after 24 h of grown in neurobasal medium for 7 days (i.e. neurosphere-forming saturated condition). Expression of CD44 (A) and Nestin (B) was detected by IIF with specific antibodies.
